# Novel perspectives on plastome evolution in Onagraceae

**DOI:** 10.1093/aobpla/plaf025

**Published:** 2025-04-24

**Authors:** Chia-Ying Ou, Chia-Hao Chang, Ting-Yu Yeh, Kuo-Fang Chung, Peter C Hoch, Shih-Hui Liu

**Affiliations:** Biological Sciences, National Sun Yat-sen University, 70 Lienhai Rd., Kaohsiung 804201, Taiwan; Science Education, National Taipei University of Education, 134, Sec. 2, Heping E. Rd.,Taipei 106320, Taiwan; Biological Sciences, National Sun Yat-sen University, 70 Lienhai Rd., Kaohsiung 804201, Taiwan; Research Museum and Herbarium (HAST), Biodiversity Research Center, Academia Sinica, Taipei 115201, Taiwan; Missouri Botanical Garden, 4344 Shaw Blvd., St. Louis, Missouri, 63110, United States; Biological Sciences, National Sun Yat-sen University, 70 Lienhai Rd., Kaohsiung 804201, Taiwan

## Abstract

Previous systematic studies have generated abundant information on plants in family Onagraceae Juss., making this taxonomic group a model for understanding plant evolution. The chloroplast genome is widely used to provide valuable insights into how plant lineages evolved. In the present study, we employed shotgun sequencing to assemble new plastomes from Onagraceae. Plastomes of ten species and one genus, *Fuchsia*, are reported for the first time. We characterize and compare the plastome features of six genera (*Chamaenerion, Circaea*, *Epilobium*, *Fuchsia*, *Ludwigia*, and *Oenothera*), allowing us to reconstruct their phylogenies and explore inter- and infra-generic evolutionary relationships, inverted repeat (IR) expansion, plastome size increases, and correlations among repeat elements, genetic variations, and evolutionary events. Our findings indicate that each of the tribes and subfamilies we assessed exhibits unique plastome features. Our phylogenetic tree supports previous findings, but also reveals that some clades need further systematic analyses. We show that increased plastome size within subfamily Onagroideae coincides with IR expansion, which is not the case for subfamily Ludwigioideae. In addition, our results indicate that higher repeat numbers and greater genetic variation can serve as indicators of evolutionary events, such as gene loss and gain, IR boundary shifts, and inversions, but they may not have arisen universally across all members of Onagraceae. Our study provides some novel insights into plastome evolution in the Onagraceae. Further studies should aim to elucidate how plastome size has evolved in Ludwigioideae and explore the evolutionary roles of regions in Onagraceae plastomes exhibiting high repeat numbers and genetic variations.

## Introduction

Plants of the evening primrose, family Onagraceae, are widely used in medicine, herbal teas, horticulture, and aquariums (e.g. Dennis 1988; Greiner and Köhl 2014; Shawky *et al.* 2021). This family currently encompasses two subfamilies, Onagroideae and Ludwigioideae, 22 genera, and approximately 670 species (Wagner *et al.* 2007). Previous studies have accumulated abundant knowledge on Onagraceae, including palynology, anatomy, cytology, embryology, reproductive biology, biochemistry, molecular phylogenies, plastomics, and paleontology (e.g. Seavey and Raven 1977a, 1977b, 1978; Raven 1979; Berry 1982; Nowicke *et al.* 1984; Tobe and Raven 1986; Conti *et al.* 1993; Hoch *et al.* 1993; Levin *et al.* 2003; Peng *et al.* 2005; Wagner *et al.* 2007; Liu *et al.* 2020; Overson *et al.* 2023). These efforts make Onagraceae an ideal group for proposing and testing various evolutionary hypotheses such as lineage origins, polyploid evolution, character evolution, shifting breeding systems, habitat preferences, speciation, and genome evolution (e.g. Eyde and Morgan 1973; Eyde 1982; Evans *et al.* 2009; Greiner *et al.* 2011; Freyman and Höhna 2019; Zupok *et al.* 2021; Liu *et al.* 2023), and thus it represents a model group for understanding plant evolution (Raven 1988; Wagner *et al.* 2007).

Molecular phylogenetics has dramatically enhanced our ability to explore evolutionary questions due to advancements in sequencing and computational techniques (Sytsma and Pires 2001; Soltis *et al.* 2018; Kress *et al.* 2022). Most plant evolutionary studies target certain DNA loci––such as the chloroplast *trn*L intron, *rps*16 intron, *trn*L-F intergenic spacer, *atp*B-*rbc*L intergenic spacer, nuclear internal transcribed spacer (ITS), and some low-copy-number nuclear genes––to reconstruct phylogenies and thereby provide evolutionary insights (e.g. Chase *et al.* 2016; Tang *et al.* 2022; Ito and Tanaka 2023; Klak *et al.* 2024). More recently, researchers have been turning to high-throughput sequencing to tackle more loci or even entire genomes for phylogenetic reconstruction, which can greatly enhance tree resolution (e.g. Jin *et al.* 2020; Romeiro-Brito *et al.* 2023; Tang *et al.* 2023). Since assembling complete chloroplast genomes is now relatively cost-effective and simple, plastomics is increasingly being applied to explore evolutionary questions (e.g. Tseng *et al.* 2022; Liu *et al.* 2024).

Previous phylogenetic studies on Onagraceae, using multiple nuclear or/and chloroplast loci, have resolved most generic relationships, yet some lower-level relationships within certain genera remain unclear (Bult and Zimmer 1993; Conti *et al.* 1993; Levin *et al.* 2003, 2004; Johnson *et al.* 2009; Overson *et al.* 2023). Recently, work by Luo *et al.* (2021) demonstrated that complete plastome data improved tree resolution at both sectional and species levels within Onagraceae, highlighting the significance of plastome data as a powerful tool for unraveling relationships and addressing evolutionary questions within this family.

Studies on chloroplast genomes in the Onagraceae were first launched in the 1930s (Renner 1934), even before DNA sequencing techniques had been developed. Early studies uncovered five different plastomes for the genus *Oenothera* L. sect. *Oenothera* subsect. *Oenothera* based on chloroplast DNA restriction site maps (Renner 1934; Stubbe 1959; Gordon *et al.* 1981, 1982). Various studies have explored plastome-nuclear genome interactions and plastome diversity in the genus *Epilobium* L. and subsect. *Oenothera* (e.g. Michaelis 1954; vom Stein and Hachtel 1986; Stubbe 1989; Greiner, Wang, Herrmann, *et al.* 2008; Greiner, Wang, Rauwolf, *et al.* 2008; Greiner *et al.* 2011; Zupok *et al.* 2021), and Schmitz and Kowallik (1986) inferred that *Oenothera* plastomes have undergone a large inversion event. These findings were built on by numerous studies on *Oenothera* plastomes (e.g. Hachtel *et al.* 1991; Greiner, Wang, Herrmann, *et al.* 2008; Greiner, Wang, Rauwolf, *et al.* 2008; Massouh *et al.* 2016; Sobanski *et al.* 2019; Zupok *et al.* 2021), yet information on the plastome evolution of other Onagraceae genera is sparse (Liu *et al.* 2018; Luo *et al.* 2021; Barloy-Hubler *et al.* 2023).

Onagraceae plastomes display a typical quadripartite structure––one large single copy (LSC), one small single copy (SSC), and two inverted repeats (IRs)––with gene content and arrangement being broadly conserved (Greiner, Wang, Herrmann, *et al.* 2008; Greiner, Wang, Rauwolf, *et al.* 2008; Liu *et al.* 2016, 2018; Yang *et al.* 2018; Luo *et al.* 2021; Barloy-Hubler *et al.* 2023; Kang *et al.* 2023). Drawing from 31 accessions representing 26 Onagraceae species across five genera (*Chamaenerion* Ség., *Circaea* L., *Epilobium*, *Ludwigia* L., and *Oenothera*), Luo *et al.* (2021) reported that Onagraceae plastomes typically contain 132 genes, with the exception being subsect. *Oenothera* in which the *ccs*A, *trn*L-UAG, *rpl*32, and *ndh*F genes are duplicated and the IR region has undergone expansion. Moreover, subsect. *Oenothera* harbors a large inversion in the LSC spanning 56 kilobases (kp), with no other large rearrangement identified in the Onagraceae (Greiner, Wang, Herrmann, *et al.* 2008; Luo *et al.* 2021). However, this information was derived from a restricted sample size from this extensively varied family. Consequently, further sampling is necessary to elucidate the variations in gene content and arrangement within Onagraceae.

Plastome sizes within the Onagraceae range from 155,817 bp (*Circaea alpina* L. subsp*. micrantha* (A.K. Skvortsov) Boufford; MZ353628) to 166,545 bp (*Oenothera grandiflora* L’Hér.; KT881173) (Massouh *et al.* 2016; Luo *et al.* 2021; Zhang *et al.* 2021). The size of the IR is expanded in the Onagraceae, with that of basal genera (*Ludwigia* and *Circaea*) containing 17 genes, those of tribes Epilobieae and Onagreae consisting of 18 genes, and those in *Oenothera* sect. *Oenothera* subsect. *Munzia* W. Dietrich encompassing 21 genes (Luo *et al.* 2021). Variations in plastome size and gene number for many plant groups have been attributed to differential IR expansion and contraction (Sun *et al.* 2016; Rabah *et al.* 2019), though tandem repeat expansion and gene/intron gain also contribute to plastome size evolution in other plant groups (Dugas *et al.* 2015; Ruhlman *et al.* 2015). Thus, further Onagraceae plastomics with additional complete plastomes would help to reconfirm the range of plastome sizes, to reexamine the IR expansion hypothesis of Luo *et al.* (2021), and to elucidate the contributors to plastome size differences in this family.

A number of studies on plastome evolution have uncovered gene loss/gain events (e.g. Jansen *et al.* 2007; Yang *et al.* 2022; Choi and Lee 2024). Two pseudogenes, *ycf*15 and *inf*A, have been uncovered in all known Onagraceae plastomes (Greiner, Wang, Rauwolf, *et al.* 2008; Luo *et al.* 2021; Barloy-Hubler *et al.* 2023), and loss of *clp*P intron 1 and 2 have been reported for subsect. *Oenothera* alone (Greiner, Wang, Rauwolf, *et al.* 2008; Luo *et al.* 2021). Gene loss/gain events can be associated with habitat adaptation, trophic modes of nutrition, speciation, IR size dynamics, and horizontal gene transfer between mitochondrial or nuclear genomes (e.g. Lin *et al.* 2017; Mohanta *et al.* 2020; Filip and Skuza 2021; Scobeyeva *et al.* 2021; Jost and Wanke 2024), and such events can promote other genetic variations (e.g. Li *et al.* 1981; Sloan *et al.* 2012; Zhu *et al.* 2016). However, previous assessments within subsect. *Oenothera* (Greiner, Wang, Rauwolf, *et al.* 2008) or Onagraceae (Luo *et al.* 2021) did not uncover significant genetic divergence in the *clp*P gene in these groups. Therefore, it remains unclear if genetic diversity in the Onagraceae plastome is generally high or whether other genes or DNA regions exhibiting relatively high genetic diversity have undergone gene loss or gain events or other forms of recombination. Moreover, sequence variations arising from the two documented *ycf*15 and *inf*A gene loss events in the Onagraceae remain unexplored. Broader sampling could enable a thorough exploration of the significance of gene loss/gain events and their associated sequence variations within the family.

Repeat elements frequently coincide with evolutionary events in plant plastomes (Ogihara *et al.* 1988; Zhang *et al.* 2019). For example, Kawata *et al.* (1997) found that palindromic repeats are involved in certain gene deletion events in many plant chloroplast genomes, and Sudianto *et al.* (2019) suggested that, in the *Lagarostrobos franklinii* (Hook.f.) Quinn plastome, rearrangements of locally collinear blocks are likely associated with repeat elements at their junctions. High frequency tandem and palindromic repeats have been identified at the margins of the 56-kb inversion in subsect. *Oenothera* (Greiner, Wang, Rauwolf, *et al.* 2008). Moreover, certain spacers and genes within *Oenothera* plastomes exhibit length variations that may be associated with repeated elements identified in those regions (Wolfson *et al.* 1991; Massouh *et al.* 2016). However, these previous studies have focused exclusively on *Oenothera*. Further studies are necessary to explore the association between repeat elements and evolutionary events in the Onagraceae.

Here, we conducted analyses of phylogenetic and plastome features to provide new perspectives on the evolutionary dynamics of Onagraceae. We had four specific goals. First, we aimed to reassess the current classifications and evolutionary relationships at both the generic and specific levels. Second, we tested the hypothesis of Luo *et al.* (2021) that early Onagraceae genera have a shorter IR than more recently evolved ones. Third, we determined if Onagraceae plastome size variations are positively related to IR size and not to gene or repeat numbers. Fourth, we examined if gene loss/gain events, IR boundaries, and large inversion borders are correlated with increased genetic variations and high repeat element numbers in these regions in Onagraceae plastomes.

## Materials and methods

### Sampling, DNA extraction, DNA library preparation, and sequencing

We assessed 36 plastomes from six genera (*Chamaenerion, Circaea*, *Epilobium*, *Fuchsia* L., *Ludwigia*, and *Oenothera*) and 31 taxa of Onagraceae, with three outgroups of family Lytrhaceae. Twelve of the plastomes have been newly assembled, and the remainder were obtained from the NCBI GenBank database (Sayers *et al.* 2022). Plant material for newly assembled sequences was obtained from fresh young leaves collected in the field or from dried leaves in the fragment packets of herbarium vouchers. Specimens of field samples have been deposited at the Herbarium of the Biodiversity Research Center, Academia Sinica, Taiwan (HAST), the Missouri Botanical Garden Herbarium (MO), and the Herbarium of Taiwan Forestry Research Institute, Taiwan (TAIF) (Thiers 2016) for further investigations. Detailed information on the sampled plastomes is provided in Table 1.

**Table 1.** Samples assessed in the present study, their GenBank accession numbers, major plastomes characteristics, and voucher information. GenBank accession numbers with asterisks are newly generated.

Scientific name	NCBI accession number	Plastome size (bp)	LSC size (bp)	SSC size (bp)	IR size (bp)	GC content (%)	Gene number (Plastome)	Gene number (LSC)	Gene number (SSC)	Gene number (IR)	Coverage of plastome	Collector, *Collection number* (Herbarium)
**Family Lythraceae**												
*Duabanga grandiflora* (Roxb. ex DC.) Walp.	NC042899	156084	86471	16501	26556	37.5	133	84	12	18 or 19		
*Punica granatum* L.	KY635883	158633	89015	18686	25466	36.9	132	84	12	18		
*Woodfordia fruticosa* (L.) Kurz	NC042898	159380	89569	18697	25557	36.6	132	84	12	18		
**Family Onagraceae**												
*Chamaenerion angustifolium* (L.) Scop.	MN481508	161182	88068	17217	27475	38.2	133	84	12	18 or 19		
*Chamaenerion angustifolium* (L.) Scop. subsp. *angustifolium*	MZ353639	160235	89062	17320	27400	33.0	133	84	12	18 or 19		
*Circaea cordata* Royle 1	OP378148*	156102	87737	18284	25040	37.8	132	85	12	17 or 18	356.9	Pi-Fong Lu, *16757* (HAST)
*Circaea cordata* Royle 2	MZ353640	156129	87766	18283	25040	32.3	132	85	12	17 or 18		
*Circaea alpina* L. subsp*. caulescens* (Kom.) Tatew.	MZ353641	156024	87659	18283	25041	37.8	132	85	12	17 or 18		
*Circaea alpina* L. subsp*. micrantha* (A.K. Skvortsov) Boufford	MZ353628	155817	87569	18256	24996	37.8	132	85	12	17 or 18		
*Circaea glabrescens* (Pamp.) Hand.-Mazz.	MZ353635	156149	87774	18271	25052	37.7	132	85	12	17 or 18		
*Circaea repens* Wall. ex Asch. & Magnus	MZ353636	156051	87688	18279	25042	37.7	132	85	12	17 or 18		
*Epilobium amurense* Hausskn. subsp. *amurense* 1	OP391238*	160562	89078	17258	27113	38.1	133	84	13	18 or 19	500.5	Ching-Sung Chang & Yu-Lan Huang, *LiuSH2104* (HAST)
*Epilobium amurense* Hausskn. subsp*. amurense* 2	MZ353631	160748	88630	17280	27419	33.2	132	83	12	18 or 19		
*Epilobium amurense* subsp. *cephalostigma* (Hausskn.) C.J. Chen, Hoch & P.H. Raven	MZ353633	161124	89163	17195	27383	38.1	133	84	12	18 or 19		
*Epilobium cylindricum* D. Don	MZ353634	160773	88681	17254	27419	38.1	133	84	12	18 or 19		
*Epilobium hirsutum* L.	MW539044	161111	89117	17310	27342	38.1	133	84	12	18 or 19		
*Epilobium minutiflorum* Hausskn.	MZ353629	160993	89145	17282	27283	38.1	133	84	12	18 or 19		
*Epilobium platystigmatosum* C.B. Rob.	OP450848*	161018	89125	17279	27307	38.1	133	84	13	18 or 19	779.0	Shu-Hui Wu, *28* (HAST)
*Epilobium royleanum* Hausskn.	MW770451	160775	88685	17252	27419	38.2	133	84	12	18 or 19		
*Epilobium sikkimense* Hausskn.	MZ353637	161144	88950	17306	27444	38.1	133	84	12	18 or 19		
*Epilobium tibetanum* Hausskn.	MZ326160	160771	88653	17280	27419	38.1	133	84	12	18 or 19		
*Epilobium ulleungensis* J. M. Chung	MH198310	160912	88915	17327	27335	38.2	133	84	13	18 or 19		
*Epilobium williamsii* P.H. Raven	MZ353630	160837	88697	17458	27341	38.1	133	84	14	17 or 18		
*Fuchsia lycioides* Andrews	OP477122*	155111	87545	18082	24742	37.7	132	85	12	17 or 18	4906.2	Paul E. Berry, *s.n.* (MO)
*Ludwigia bonariensis* (Micheli) H. Hara	OP574197*	159615	90291	19800	24762	42.2	132	85	13	17	946.4	Shih-Hui Liu, *2012GH13* (MO)
*Ludwigia decurrens* Walter	OP499828*	159210	89908	19806	24748	37.4	132	84	13	17	1486.7	Shih-Hui Liu, *1858* (MO)
*Ludwigia erecta* (L.) H. Hara	OP537027*	158233	89200	19509	24762	37.4	132	85	13	17	1077.0	Shih-Hui Liu & Chia-Hao Chang, *2109* (TAIF)
*Ludwigia hyssopifolia* (G. Don) Exell	OQ220351*	158356	89326	19534	24748	37.4	132	84	13	17 or 18	2045.8	Shih-Hui Liu & Chia-Hao Chang, *2223* (TAIF)
*Ludwigia lagunae* (Morong) H. Hara	OQ024228*	159611	90297	19802	24756	37.3	132	85	13	17	1271.1	Shih-Hui Liu, *2014GH90* (MO)
*Ludwigia microcarpa* Michx.	OP485125*	158912	89619	19549	24872	37.3	132	85	13	17	1234.8	Ching-I Peng, *18507* (MO)
*Ludwigia octovalvis* (Jacq.) P.H. Raven	KX827312	159396	90183	19703	24755	37.4	132	85	13	17	198.0	Shih-Hui Liu, *2014GH96* (MO)
*Ludwigia perennis* L.	OP537026*	158665	89620	19493	24776	37.3	132	85	13	17	1794.8	Tsai-Wen Hsu, *18907* (TAIE)
*Ludwigia sedoides* (Bonpl.) H. Hara	OP574196*	159585	90201	19814	24785	42.1	132	85	13	17	488.5	Peter C. Hoch, *s.n.* (MO)
*Oenothera biennis* L.	NC010361	164807	88964	18091	28471	39.1	133	85	13	17 or 18		
*Oenothera curtiflora* W.L. Wagner & Hoch	MT726052	161318	89133	17383	27401	34.0	132	84	11	18 or 19		
*Oenothera picensis* Phil. subsp*. picensis*	KX118607	167092	88323	16491	31139	35.1	137	85	9	21 or 22		

Genomic DNA from samples for newly assembly was isolated using a DNeasy Plant Pro Kit (Qiagen, Hilden, Düsseldorf, Germany). Referring to previous studies on *Ludwigia* (Liu *et al.* 2017, 2018), we optimized the isolation protocol by applying a 30-minute incubation period at 65°C for cell lysis to enhance DNA product. Genomic DNA quality and quantity were checked using an Invitrogen Qubit 4 Fluorometer (Thermo Fisher Scientific, Waltham, Massachusetts, USA) (Qubit 4) and 1.5% agarose gel electrophoresis.

If the genomic DNA was longer than 400-500 bp, we conducted DNA shearing using a Bioruptor Pico Sonication System (Diagenode, Liège, Belgium). Next, a NEBNext Ultra™ DNA Library Prep Kit for Illumina (New England Biolabs, Ipswich, MA, USA) was used to prepare and optimize the DNA library. Qubit 4, 1.5% agarose gel electrophoresis, and an Agilent 5300 Fragment Analyzer System (Agilent Technologies, Santa Clara, California, USA) were applied to validate the quality and quantity of DNA libraries. Validated DNA libraries were then pooled and sequenced commercially by Genomics BioSci & Tech. Co. Ltd. (New Taipei City, Taiwan) using a NovaSeq 6000 System (Illumina, San Diego, California, USA) to generate 150-bp paired-end reads.

### Plastome assembly and annotation

Quality control of the resulting Illumina reads was conducted in Falco 1.2.1 (Andrews 2010; de Sena Brandine and Smith 2021) with default parameters. Adapters and low-quality bases were trimmed from both ends utilizing the BBDuk tool in BBMap 39.01 (Bushnell 2014) with parameters k = 27, hdist = 1, trimq = 10, qtrim = rl, minlength = 30, and trimpolyg = 6. The plastomes of our samples were assembled according to Liu *et al.* (2018) using Geneious Prime 2023.2.1 (https://www.geneious.com). Published plastomes of *L. octovalvis* (Jacq.) P.H. Raven (KX827312) and *E. ulleungensis* J. M. Chung (MH198310) were applied as references on initiation of assembly. Protein coding genes, exons, introns, tRNAs, rRNAs, as well as the LSC, SSC, and IRs of the newly assembled plastomes were annotated by utilizing CPGAVAS2 (Shi *et al.* 2019) with a default setting and by transfer from the two aforementioned reference plastomes in Geneious Prime 2023.2.1 with 85% or higher similarity. Annotated protein coding, tRNA, and rRNA genes were validated in BLAST + 2.14.0 (Camacho *et al.* 2009), tRNAscan-SE 2.0 (Chan and Lowe 2019), and RNAmmer 1.2 (Lagesen *et al.* 2007), respectively, with default settings. Finally, the complete, annotated plastomes were depicted using CPGAVAS2 (Shi *et al.* 2019) and OGDRAW 1.3.1 (Greiner *et al.* 2019).

### Phylogenetic tree reconstruction

Aligning nonhomologous sequences can be problematic for subsequent phylogenetic analysis (Kapli *et al.* 2020; Edgar 2022; Emms and Kelly 2022). To acquire a highly accurate alignment, we individually aligned the sequences of each gene––including protein-coding genes, tRNA genes, and rRNA genes, and their coding sequence and introns––and then the intergenic spacers of the sampled plastomes were extracted and aligned individually. Each annotated gene and pseudogene was extracted using Geneious Prime 2023.2.1 and they were aligned separately in MUSCLE 5 (Edgar 2022). To rapidly identify introns and intergenic spacers, we used MAFFT v7.490 (Katoh *et al.* 2002; Katoh and Standley 2013) to generate an a priori alignment of the annotated plastomes. Using this a priori alignment (abbreviated hereafter as pAL) as an escort, we extracted the sequences of each intron and intergenic spacer from Geneious Prime 2023.2.1 and then realigned them individually using MUSCLE 5 (Edgar 2022). The genes and intergenic spacers in IR regions were aligned and applied to subsequent phylogenetic analysis only once. Later, all gene and intergenic spacer alignments were concatenated into a final alignment (abbreviated hereafter as fAL).

Ignoring compositional heterogeneity within genomic data can result in incorrect phylogenetic inferences (Kainer and Lanfear 2015; Walker *et al.* 2019; Steenwyk *et al.* 2023). We estimated the best-fit substitution model of each gene and intergenic spacer using PartitionFinder 2 (Lanfear *et al.* 2017) implanted in IQ-tree 2.2.2.7 (Minh *et al.* 2020). Phylogenetic trees based on the final alignment and the best-fit substitution models were reconstructed by applying maximum likelihood (ML) and Bayesian inference (BI) algorithms using IQ-tree 2.2.2.7 (Minh *et al.* 2020) and MrBayes 3.2.7a (Ronquist and Huelsenbeck 2003; Ronquist *et al.* 2012), respectively, in the CIPRES Science Gateway (Miller *et al.* 2010). To generate the ML tree, 100 replicates of tree searches were conducted first. Then, 1,000 bootstrap (bs) iterations were applied to evaluate branch supports for the ML tree. To generate the BI tree, two individual runs of 5 × 10^6^ generations were performed, one tree was sampled every 1,000 generations, and the posterior probabilities (pp) on the 50% majority rule consensus BI tree were summarized with 0.25 burn-in. Topology conflict between ML and BI trees was examined using the Shimodaira-Hasegawa (SH) (Kishino and Hasegawa 1989) and Kishino-Hasegawa (KH) (Kishino and Hasegawa 1989) tests in PAUP* 4.0a (Swofford 2003). Finally, FigTree 1.4.4 (Rambaut 2018) was employed to depict the ML and BI trees.

### Plastome feature analyses and comparisons

Guided by our phylogeny, the following plastome features were analyzed to understand the plastome evolution in Onagraceae.

Numbers of genes, pseudogenes, and introns were confirmed in Geneious Prime 2023.2.1. The sizes and boundaries of the quadripartite structure for sampled taxa were analyzed using IRscope (Amiryousefi *et al.* 2018) and confirmed in Geneious Prime 2023.2.1. Pearson correlation analyses were conducted to test our hypothesis concerning the relationship between plastome size and IR size or gene number. Large-scale rearrangements and inversions in the Onagraceae plastomes were detected by identifying and aligning local collinearity using MAUVE 1.1.3 (Darling *et al*. 2004, 2011) implanted in Geneious Prime 2023.2.1 and by performing local and global alignments using mVISTA (Frazer *et al.* 2004). The pairwise syntenic dotplots between paired taxa were also generated by applying mVISTA (Frazer *et al.* 2004).

Moreover, to explore genetic variations among the taxa we sampled, we calculated nucleotide variations (Pi; π; Nei 1987) and sequence identities. The π along the plastomes of sampled genera, subfamilies, and Onagraceae were determined using a sliding window analysis in DNASP 6 (Rozas *et al.* 2017) with a step size of 50 bp and a 200-bp window based on pAL. Average pairwise sequence identities (abbreviated hereafter as APSI) were also computed in Geneious Prime 2023.2 to cover gaps skipped by the sliding window analysis, as well as to provide insights into each gene and spacer of Onagraceae plastomes. Gene and spacer alignments generated previously for phylogenetic tree reconstruction were applied for the APSI analysis.

In addition, repeat elements and their locations on the plastomes were detected using the following three implants in CPGAVAS2 (Shi *et al.* 2019). Microsatellites (SSRs) with motif sizes of one to six were identified using default settings in MISA-web (Beier *et al.* 2017). Tandem repeats were searched using Tandem Repeats Finder 4.09 (Benson 1999) with parameters 2 7 7 80 10 50 2000 -f -d -m. Dispersed and palindromic repeats were identified by applying default parameters in Vmatch 2.3.1 (Kurtz 2016). Repeat elements located in IR regions were counted only once. When repeat elements overlapped, only the longest one was counted. Pearson correlation analyses were applied to investigate correlations between plastome sizes and repeat numbers and to test our hypothesis that plastome size variations are positively related to IR size.

## Results

### Plastome assembly and annotation

**Figure 1.**
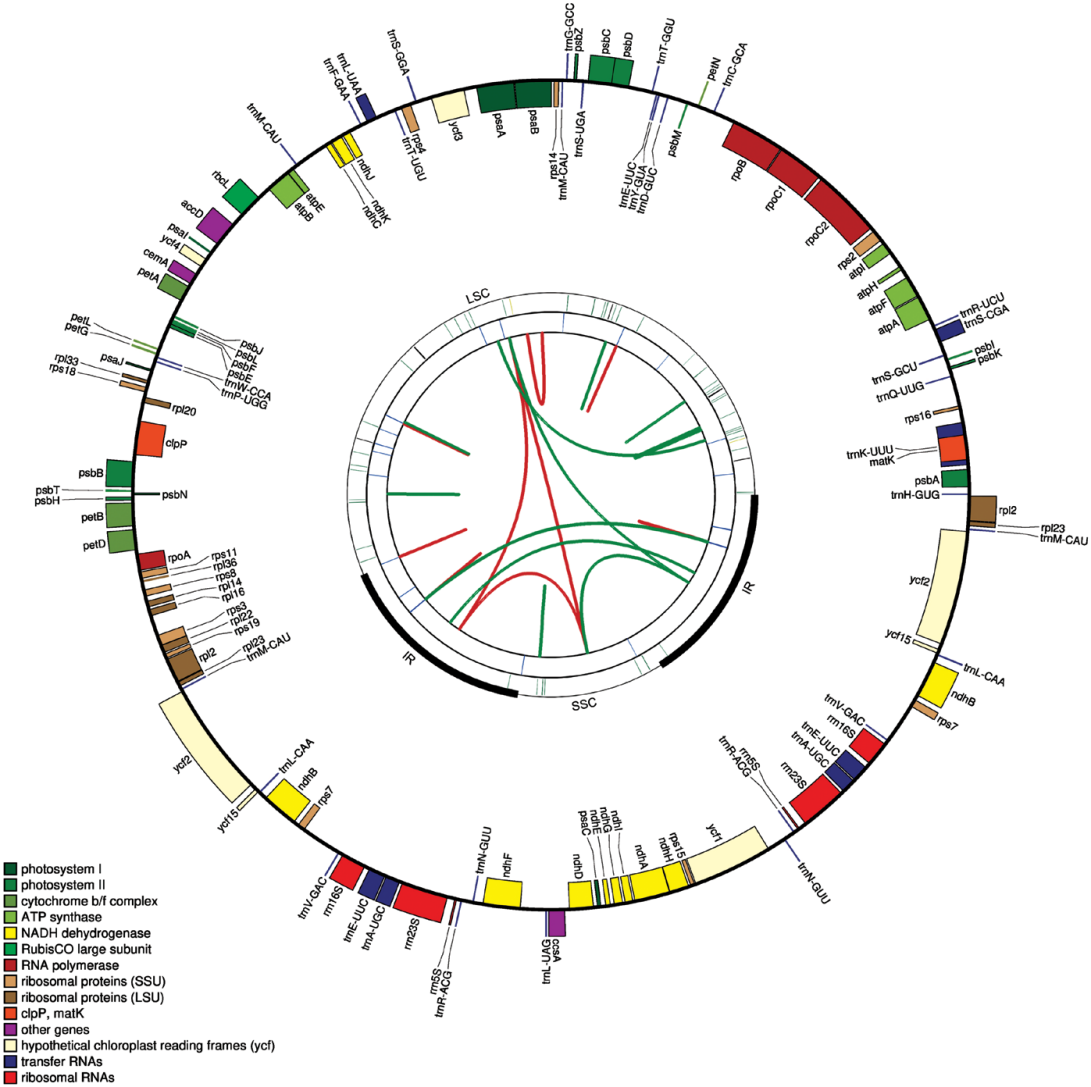
Summary of the complete chloroplast genome of Onagraceae (155,111 bp – 167,092 bp) using *Fuchsia lycioides* as a basis. The four rings from outside to inside show the locations of genes, microsatellites, tandem repeats, and forward (red) and reverse (green) repeats, respectively. Genes are color-coded according to their function, as per the legend. Genes inside the first ring are in clockwise directions, whereas genes on the outside are in counterclockwise directions. Large single copy (LSC), small single copy (SSC), and inverted repeat (IR) regions are annotated on the second ring.

We have newly sequenced, assembled, and annotated 12 Onagraceae plastomes. To the best of our knowledge, the plastomes of one genus, *Fuchsia*, and ten species––namely *E. platystigmatosum* C.B. Rob., *F. lycioides* Andrews, *L. bonariensis* (Micheli) H. Hara, *L. decurrens* Walter, *L. erecta* (L.) H. Hara, *L. hyssopifolia* (G. Don) Exell, *L. lagunae* (Morong) H. Hara, *L. microcarpa* Michx., *L. perennis* L., and *L. sedoides* (Bonpl.) H. Hara—have not been reported previously. All 12 newly generated plastomes show the typical quadripartite structure with lengths of 155,111 bp to 161,018 bp and contain 132 to 133 unique genes (Figs. 1, Supporting Information File S1, Table 1). Average coverages of these plastomes ranged from 357 × to 4,906 × (Table 1).

The newly assembled plastomes have been deposited to GenBank (Sayers *et al.* 2019), with respective accession numbers presented in Table 1.

### Phylogenetic analysis

In our a priori alignment (or pAL), we detected an expected large-scale inversion in *O. biennis* L., with this 56-kb inversion occurring in the taxa of subsect. *Oenothera* (Greiner, Wang, Herrmann, *et al.* 2008; Greiner, Wang, Rauwolf, *et al.* 2008), with no other large-scale rearrangements detected for the remaining Onagraceae that we examined. Further details of this large-scale rearrangement are presented below. To extract the introns and intergenic spacers correctly, we modified the *O. biennis* plastome by reversing its 56-kb inversion and performed the alignment again using the modified plastome (abbreviated hereafter as mpAL).

**Figure 2.**
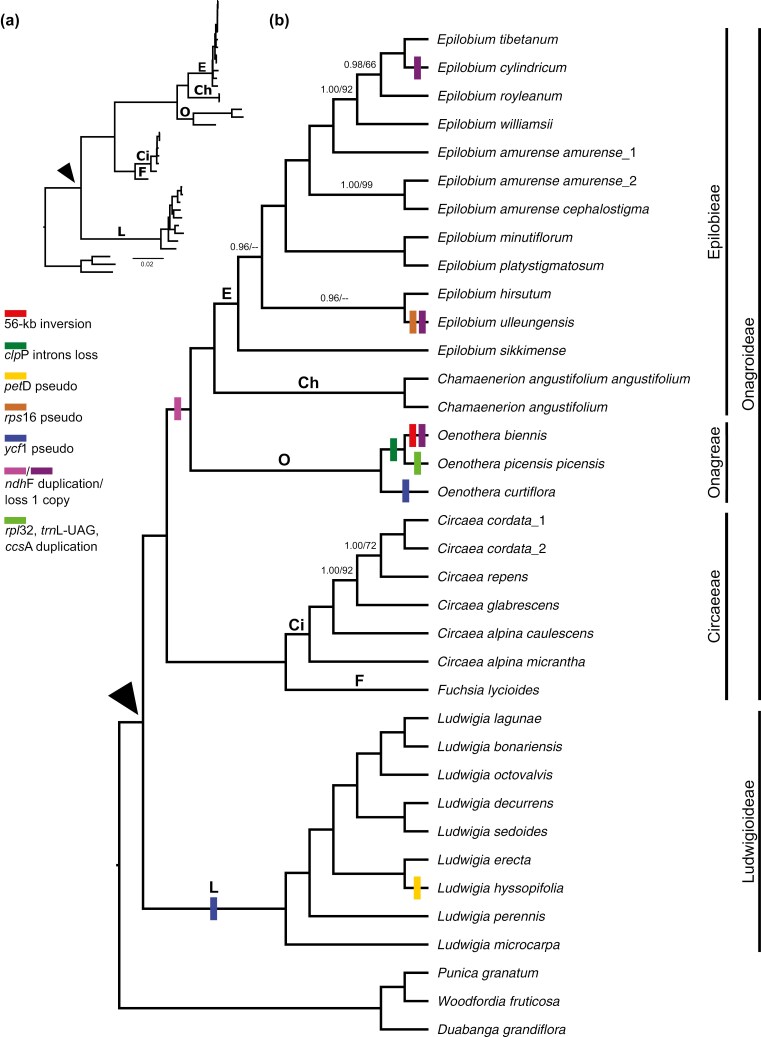
Bayesian 50% majority rule consensus tree based on 33 Onagraceae and three Lythraceae plastomes. The arrow indicates the crown node of Onagraceae. Ch, Ci, E, F, L, and O denote the crown nodes of *Chamaenerion*, *Circaea*, *Epilobium*, *Fuchsia*, *Ludwigia*, and *Oenothera*, respectively. In cases where branches are not fully supported, posterior probabilities (pp)/bootstrapping (bs) values are shown. Hyphens indicate if the pp or bs value is less than 0.70 or 50, respectively. Bars on the tree indicate gene or intron loss and regain events. Vertical lines at the right of the tree indicate the two Onagraceae subfamilies and tribes of Onagroideae. Branch lengths are shown in (a) and Supporting Information File S2, and have been transformed in (b). The scale bar denotes the branch length.

Our BI and ML trees share the same topology. Both the SH and KH tests support topological consistency between the BI and ML trees (*p*-value > 0.05; Supporting Information File S2). Accordingly, only the BI tree topology is presented herein (Fig. 2 and Supporting Information File S3). The final alignment (or fAL), partition schemes, and BI and ML tree files are given in Supporting Information File S4. Our trees indicate that all of the Onagraceae genera we studied form well-supported monophyletic groups (bs = 100, pp = 1.00). In our trees, *Ludwigia* is the basal branch of Onagraceae, *Circaea* and *Fuchsia* are sister clades, *Chamaenerion* is sister to *Epilobium*, and *Oenothera* is sister to *Chamaenerion *+* Epilobium*. Infrageneric relationships of the studied genera are well resolved, except for a few branches in *Epilobium*. More specifically, in Fig. 2, though *E. sikkimense* Hausskn. is the basal branch of all studied *Epilobium* taxa and *E. hirsutum* L. is sister to *E. ulleungensis*, support for the relationships among these three taxa is weak (bs < 50, pp = 0.96). Moreover, the relationship between *E. royleanum* Hausskn. and *E. cylindricum* D. Don + *E. tibetanum* Hausskn. is not firmly supported (bs = 66, pp = 0.98).

### Plastome size and quadripartite structure

Our analyses of 33 Onagraceae taxa reveal that plastome size ranges from 155,111 bp to 167,092 bp, the LSC ranges in size from 87,545 bp to 90,297 bp, the SSC ranges from 16,491 bp to 19,814 bp in length, and the IR ranges from 24,742 bp to 31,139 bp (Tables 1-2). Compared to other families in the order Myrtales, Onagraceae have medium-sized plastomes (Table 2). Within Onagraceae, *Oenothera* appears to have the largest plastome, LSC, and IR, but the smallest SSC. *Fuchsia* has the smallest plastome, LSC, and IR, while *Circaea* possesses the largest SSC in the Onagraceae we examined. Plastome, LSC, SSC, and IR sizes within a genus vary and are not related to the sample size of each genus, but are to plastome characteristics. The size variations we detected for *Oenothera* are considerably larger than those observed for other studied genera, with tribe Circaeeae demonstrating the smallest size variations within Onagraceae. Pearson correlation analyses revealed that Onagraceae plastome size is significantly and positively correlated to IR size (r = 0.8548; Table 3). This correlation is primarily driven by Onagroideae (Table 3).

**Table 2.** Plastome, LSC, SSC, and IR sizes of Onagraceae, as well as for selected other families in Order Myrtales. Numbers of genes in each plastome region for the genera in Onagraceae studied herein are shown in square brackets.

	Sample size	Plastome size (bp) [No. genes]	LSC (bp) [No. genes]	SSC (bp) [No. genes]	IR (bp) [No. genes]	Reference
Family Onagraceae	33	155,111–167,092 Mean 159,648	87,545–90,297 Mean 88,872	16,491–19,814 Mean 18,143	24,742–31,139 Mean 26,304	This study
Subfamily Ludwigioideae						
*Ludwigia*	9	158,233–159,615 [132]	89,200–90,297 [84–85]	19,493–19,814 [13]	24,748–24,872 [17 – 17 or 18]	This study
Subfamily Onagroideae						
Tribe Circaeeae						
*Fuchsia*	1	155,111 [132]	87,545 [85]	18,082 [12]	24,742 [17 or 18]	This study
*Circaea*	6	155,817–156,149 [132]	87,569–87,774 [85]	18,256–18,284 [12]	24,996–25,052 [17 or 18]	This study
Tribe Epilobieae						
*Chamaenerion*	2	160,235–161,182 [133]	88,068–89,062 [84]	17,217–17,320 [12]	27,400–27,475 [18 or 19]	This study
*Epilobium*	12	160,562–161,144 [133]	88,630–89,163 [84]	17,195–17,458 [12–14]	27,113–27,444 [18 or 19]	This study
Tribe Onagreae						
*Oenothera*	3	161,318–167,092 [132–137]	88,323–89,133 [84–85]	16,491–18,091 [9–13]	27,401–31,139 [17 or 18 – 21 or 22]	This study
Family Lythraceae	22	152,049–160,769	83,817–89,569	16,501–33,301	17,541–26,907	(Gu *et al.* 2019)
Family Melastomataceae	42	159,995	85,754	16,984	26,888	(Zhang *et al.* 2021)
Family Myrtaceae	19	159,583	88,310	18,596	26,339	(Zhang *et al.* 2021)
Family Vochysiaceae	7	164,202	90,171	16,673	29,036	(Zhang *et al.* 2021)
Family Combretaceae	8	159,047	88,113	18,269	26,334	(Zhang *et al.* 2021)

**Table 3.** Pearson correlations for plastome features in Onagraceae. Asterisks denote correlations with p-values less than 0.05.

Features	Correlation (+/– r)	p-value
Plastome sizes vs. IR sizes		
Family Onagraceae	+ 0.8548	2.44E-10 *
Subfamily Onagroideae	+ 0.9843	4.51E-18 *
Tribe Circaeeae	+ 0.9871	3.62E-05 *
Tribe Epilobieae	+ 0.2250	0.4392
Tribe Onagreae	+ 0.9353	0.2303
Subfamily Ludwigioideae	– 0.0481	0.9022
Plastome sizes vs. gene numbers		
Family Onagraceae	+ 0.7597	2.94E-07 *
Subfamily Onagroideae	+ 0.7760	8.35E-06 *
Tribe Circaeeae	+ 0.1386	0.7670
Tribe Epilobieae	+ 0.1357	0.6438
Tribe Onagreae	+ 0.8990	0.2886
Subfamily Ludwigioideae	+ 0.0050	0.9899
Plastome sizes vs. repeat numbers		
Family Onagraceae	+ 0.7212	2.19E-06 *
Subfamily Onagroideae	+ 0.7706	1.06E-05 *
Tribe Circaeeae	– 0.5057	0.2469
Tribe Epilobieae	+ 0.2594	0.3704
Tribe Onagreae	+ 0.5368	0.3693
Subfamily Ludwigioideae	+ 0.5797	0.1018

The plastomes of Onagraceae contain 114 to 116 unique genes and 132 to 137 genes in total (Tables 1-2). All unique genes and their functions are listed in Supporting Information File S5. *Oenothera* has more genes in its IR (up to 22 genes) and in its overall plastome (up to 137 genes) compared to other genera in the Onagraceae. In general, the genera possessing larger plastome, SSC, and IR sizes have higher numbers of genes in the respective regions. Again, gene number variation among taxa of *Oenothera* is remarkably greater than for other genera in the Onagraceae. Plastome sizes of Onagraceae and Onagroideae are significantly and positively correlated with their respective gene numbers (r = 0.7597 and 0.7760 respectively; Table 3).

### Gene and intron loss/gain events

We detected several gene and intron loss events in the Onagraceae. Introns of the *clp*P gene have been lost in *O. biennis* and *O. picensis* Phil. subsp*. picensis*, both of which are classified in sect. *Oenothera*. The *pet*D pseudogene was detected in *L. hyssopifolia* alone, and the *rps*16 pseudogene was only observed in the *E. ulleungensis* plastome. The *ycf*1 gene is absent from all *Ludwigia* species we examined and from *O. curtiflora* W.L. Wagner & Hoch. Furthermore, along with IR expansion (Tables 1-2), all studied taxa of *Chamaenerion*, *Epilobium*, and *Oenothera* possess a duplicated *ndh*F gene, except for *E. cylindricum*, *E. ulleungensis*, and *O. biennis*. *Epilobium ulleungensis* and *O. biennis* have relatively large IR regions but only one *ndh*F gene. *Epilobium cylindricum* has two copies of the *ndh*F gene, only one of which is functional since the other has a stop codon inserted within it. Possessing the largest IR of the Onagraceae taxa we studied, *O. picensis* subsp*. picensis* has three duplicated genes, i.e. *rpl*32, *trn*L-UAG, and *ccs*A. These gene/intron loss or duplication events are depicted in Fig. 2.

### IR boundary and gene order arrangement

**Figure 3.**
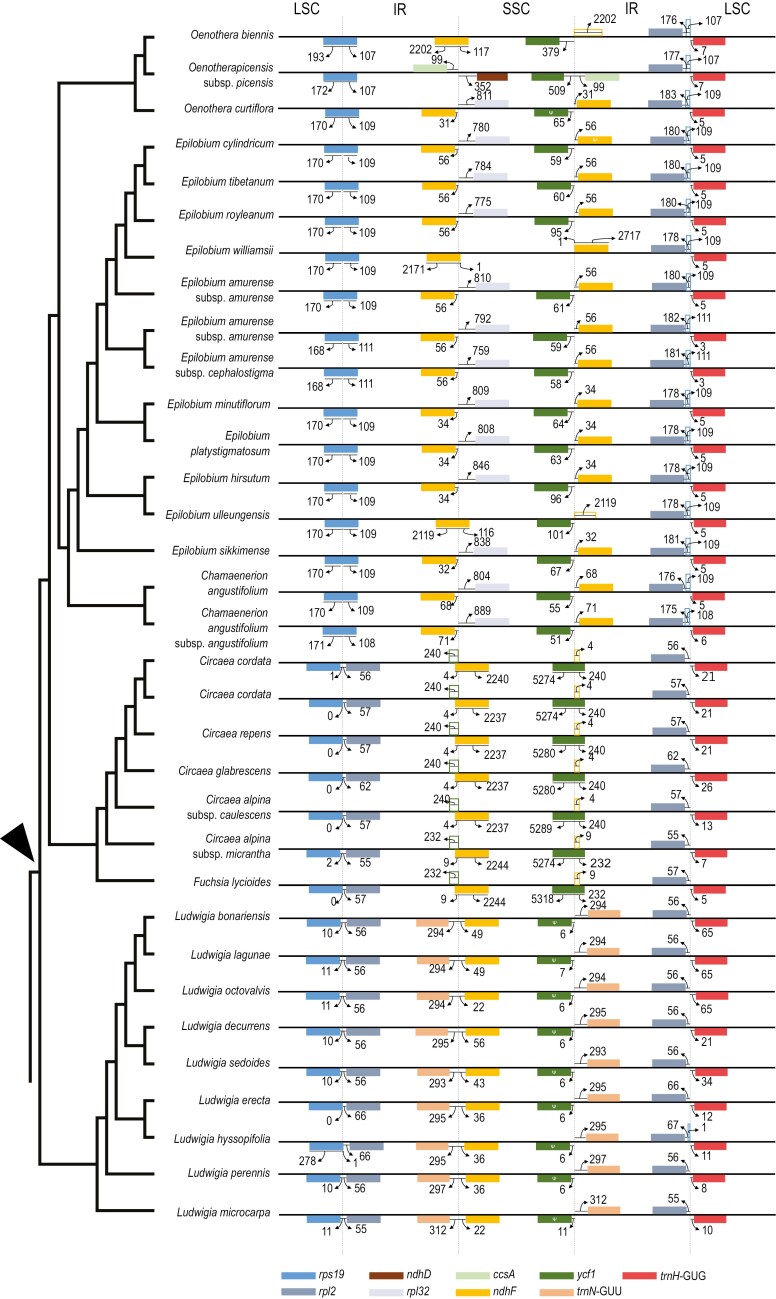
Comparisons of the boundaries of the large single copy (LSC), small single copy (SSC), and inverted repeat (IR) regions of plastomes in the six genera of the Onagraceae in this analysis indicate that each genus displays a unique pattern with a few variations. The phylogenetic tree in the left panel refers to Fig. 2, and the arrow indicates the crown node of the Onagraceae. Thick black lines represent the plastome sequences. Genes near the boundaries are shown on the plastome sequences as colored solid boxes, whereas pseudogenes are shown as colored open boxes or ψ. Thin arrows with numbers denote the lengths between region boundaries and the start or end of genes/pseudogenes.

In addition, analysis of the boundaries of the quadripartite structure (Fig. 3) indicates that each genus displays a unique pattern with a few variations, but with *Oenothera* notably varying and showing at least three patterns. Moreover, we observed that the IR of the Onagraceae has expanded more into the SSC than the LSC.

Our MAUVE and mVISTA analyses revealed that gene order arrangements in the Onagraceae are conserved, except for *O. biennis*, which has a large 56-kb inversion in the LSC between the *rbc*L and *trn*Q-UUG genes (Supporting Information Files S6). No further gene order rearrangements were detected.

### Genetic variations vs. evolutionary events

**Figure 4.**
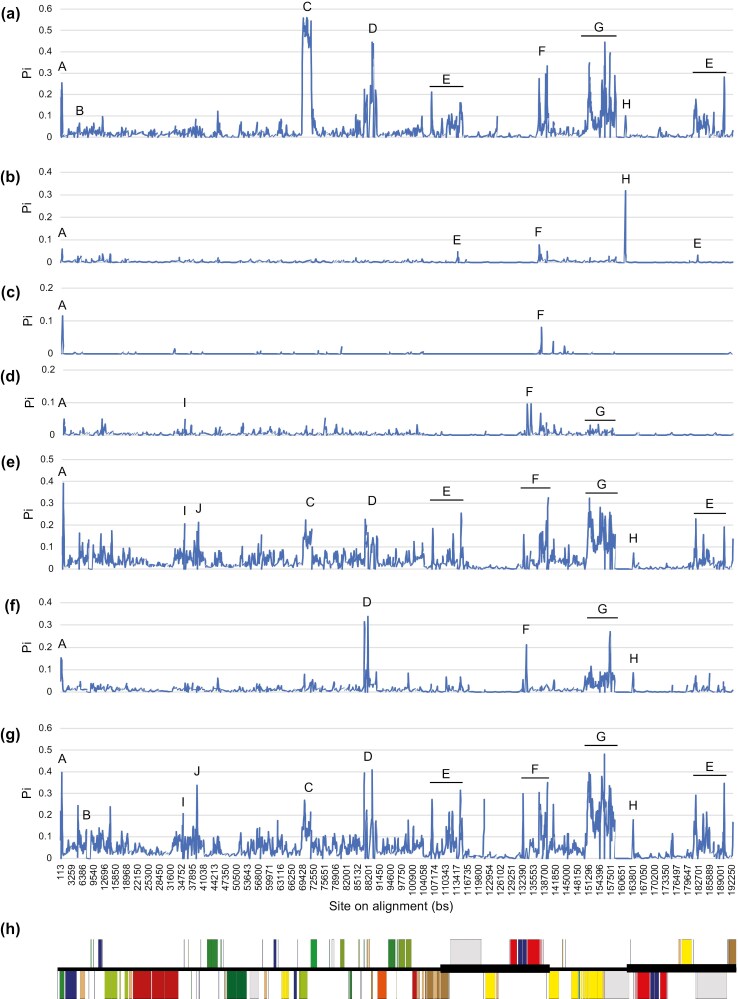
Nucleotide variations (Pi; π) for plastomes within (a) *Oenothera*, (b) *Epilobium*, (c) *Chamaenerion*, (d) *Circaea*, (e) subfamily Onagroideae, (f) *Ludwigia* or subfamily Ludwigioideae, and (g) family Onagraceae based on the a priori alignment with the modified plastome (mpAL), in which the 56-kb inversion of *Oenothera biennis* has been inverted. The complete chloroplast genome of *Fuchsia lycioides* (h) is shown here as a reference to indicate the positions and directions of genes on the mpAL, with genes color-coded as in Fig. 1. Eleven regions with high π are annotated with letters. Genes, spacers, quadripartite structure boundaries, and evolutionary events found in these regions are listed in Table 4.

Upon applying the pAL in our sliding window analyses, π within the 56-kb inversion of *O. biennis* are extremely high (Supporting Information File S7), but then details for π in Onagroideae and in all Onagraceae within this region were lost. Therefore, as an alternative approach, we applied the mpAL so details of π within this region could be presented accurately. Our results reveal ten regions of high π in Onagraceae plastomes (Fig. 4g).

Our sliding window analyses (Fig. 4; Table 4) show that two of the regions of high π (F and H) are located at the boundaries of IR and SSC regions, with π for the IR-SSC boundary being generally higher than that of the SSC-IR boundary. Both the F and H regions were detected in all studied genera, except for *Chamaenerion* and *Circaea*, neither of which possessed the H region. Unfortunately, the boundaries of the IR and LSC regions were not detected as having high π for any of the studied genera because of the gaps skipped by the sliding window analysis. High π for the borders of the 56-kb inversion in *O. biennis* (B and C regions) were determined only for *Oenothera* and for higher-level groupings that included *Oenothera*. Moreover, the *clp*P, *ycf*1, and *ycf*2 genes (D, G, and E regions, respectively) displayed high π. *Oenothera* and *Ludwigia* possess all three of these regions, whereas *Circaea* has only the G region and *Epilobium* only the E region. Additionally, three spacers––*trn*H-GUG-*psb*A, *pet*N-*psb*M, and *trn*E-UUC-*trn*T-GGU (A, I, and J regions, respectively)––presented high peaks in our sliding window analysis. All of the genera we studied have the A region. The I region showed high π peaks only in *Circaea*, Onagroideae, and Onagraceae, and the J region was detected only in Onagroideae and Onagraceae groupings. Generally, plastomes within *Chamaenerion*, *Circaea*, and *Epilobium* displayed relatively low π, whereas those in *Oenothera* had high π.

**Table 4.** Seventeen regions with low average pairwise sequence identities (APSI) and/or high nucleotide variation (Pi; π) identified in Onagraceae plastomes. Genes, spacers, quadripartite structure boundaries, and evolutionary events located in these regions are indicated. The ‘V’ denotes regions with low APSI and/or high π detected within the clade. The sample size for each genus is indicated in parentheses.

		Subfamily Ludwigioideae		Subfamily Onagroideae										Family	
				Tribe Circaeeae				Tribe Epilobieae				Tribe Onagreae			
		Ludwigia		Circaea		Fuchsia		Chamaenerion		Epilobium		Oenothera		Onagraceae	
		(9)		(6)		(1)		(2)		(12)		(3)		(33)	
Region	Gene or spacer	π	APSI	π	APSI	π	APSI	π	APSI	π	APSI	π	APSI	π	APSI
A	*trn*H-GUG-*psb*A spacer	V	V	V	V			V	V	V	V	V	V	V	V
A’	*rpl*2-*trn*H-GUG spacer (IR-LSC boundary)		V		V						V				V
B	*rps*16-*trn*Q-UGG spacer (border of the large 56-kb inversion in *Oenothera biennis*)		V									V	V	V	V
C	*acc*D or *rbc*L-*acc*D spacer (border of the large 56-kb inversion in *Oenothera biennis*)		V									V	V	V	V
D	*clp*P	V	V									V	V	V	V
D’	*rps*12 exon 1-*clp*P spacer		V										V		V
E	*ycf*2									V	V	V	V	V	V
E’	*trn*I-CAU-*ycf*2 spacer												V		V
F	*ndh*F-*rpl*32 spacer (IR-SSC boundary)	V	V	V	V			V	V	V	V	V	V	V	V
F’	*rpl*32-*trn*L-UAG spacer				V				V						V
G	*ycf*1	V	V	V								V	V	V	V
H	*trn*N-GUU-*ndh*F spacer (SSC-IR boundary)	V	V							V		V		V	
I	*pet*N-*psb*M spacer			V	V				V		V		V	V	V
J	*trn*E-UUC-*trn*T-GGU spacer													V	V
K	*rpl*22-*rps*19 spacer or *rps*19-*rpl*2 spacer (LSC-IR boundary)												V		V
L	*trn*R-UCU-*atp*A spacer		V								V				V
M	*ccs*A-*ndh*D spacer		V		V						V				V

**Figure 5.**
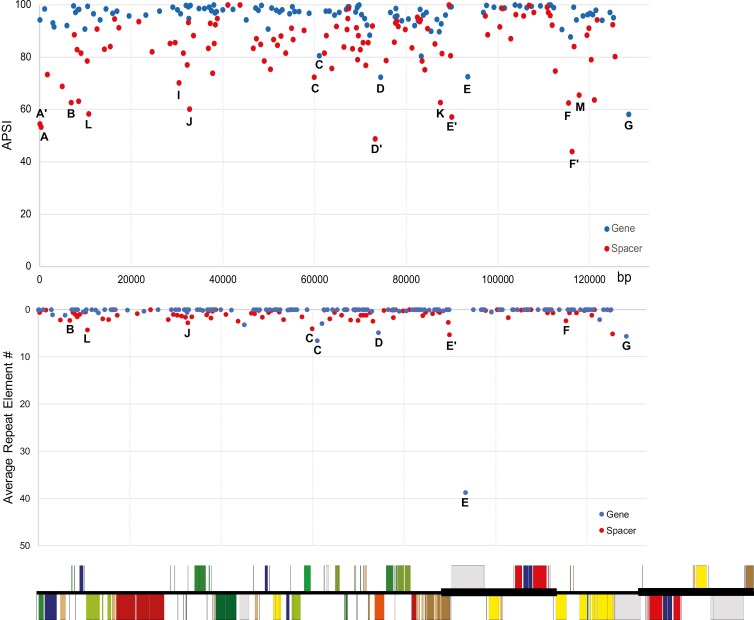
(a) Average pairwise sequence identities (APSI) and (b) average repeat numbers for each chloroplast gene and spacer in Onagraceae. Genes or spacers with lower APSI are denoted with letters. The positions of each gene and spacer on the complete chloroplast genome (relative to the *Fuchsia lycioides* plastome shown at the bottom) are shown along the x-axis. Note that only one reverted repeat is displayed in (a) and (b). Regions with low APSI or high average repeat numbers are annotated with letters. Genes, spacers, quadripartite structure boundaries, and evolutionary events found in these regions are listed in Table 4.

Similar to the results of our sliding window analyses, our APSI analyses indicate that each genus presents a unique pattern of genetic variations (Table 4; Fig. 5). The APSI analysis not only validated the regions of high π of our sliding window analysis, but also identified additional genes and spacers exhibiting low genetic variation. For example, the IR-LSC (A’ region) and LSC-IR (K region) boundaries were detected in the APSI analyses. The B region was apparent not only in *Oenothera*, but also in *Epilobium* and *Ludwigia*. Moreover, we detected three low APSI spacers––*rps*12 exon 1-*clp*P spacer (D’ region), *trn*I-CAU-*ycf*2 spacer (E’ region), and *rpl*32-*trn*L-UAG spacer (F’ regions)––proximal to regions of high π (D, E, and F regions, respectively). Two additional low APSI regions, *ccs*A-*ndh*D spacer (M region) and *trn*R-UCU-*atp*A spacer (L region), were observed in several genera of the Onagraceae.

Next, we conducted a comparative APSI analysis to explore intron or gene loss and duplication events. *Ludwigia* and *Oenothera* displayed lower APSI in the *ycf*1 gene than other genera, which is consistent with *ycf*1 pseudogenization events occurring in *Ludwigia* and *Oenothera* (Fig. 2; Table 4). Moreover, our results from the *ndh*F gene indicate that the tribe hosting two copies of this gene, i.e. tribe Epilobieae, has a higher APSI than other tribes only having one copy (Fig. 2; Table 4). Other loss or duplication events are too sparse in our phylogeny to examine changes further by APSI.

### Repeats vs. evolutionary events

On average, we detected 61 SSR, 25 tandem, 11 dispersed, and seven palindromic repeats among Onagraceae plastomes (Table 5; Supporting Information Files S8-S9). Overall, the types, numbers, and distributions of repeat elements varied considerably among genera in the family. Repeat numbers were significantly and positively correlated with plastome size in Onagraceae and in Onagroideae, with values of Pearson correlation r = 0.7212 and 0.7706, respectively (Table 3), and they also exhibited strong correlation with our APSI data (Fig. 5; Supporting Information Files S8-S9). In the Onagroideae, tribe Circaeeae plastomes harbor fewer repeat elements than those of either Epilobieae or Onagreae (Table 5; Supporting Information File S8). Interestingly, earlier branching genera in the Onagroideae tended to have fewer SSRs, tandem repeats, and dispersed repeats, yet more palindromic repeats, than more recently evolved ones. Ludwigioideae plastomes have more palindromic repeats but fewer SSRs than Onagroideae.

**Table 5.** Average numbers of repeat elements in the plastome of the Onagraceae. The sample size of each genus is shown in parentheses.

	Subfamily Ludwigioideae	Subfamily Onagroideae					Family
		**Tribe Circaeeae**		**Tribe Epilobieae**		**Tribe Onagreae**	
	***Ludwigia***	***Circaea***	***Fuchsia***	***Chamaenerion***	***Epilobium***	***Oenothera***	**Onagraceae**
	(9)	(6)	(1)	(2)	(12)	(3)	(33)
Average no. microsatellites	35.2	50.7	55.0	84.5	79.8	65.3	60.5
Average no. tandem repeats	30.8	18.0	17.0	31.5	19.8	39.3	24.8
Average no. dispersed repeats	13.1	7.0	9.0	20.5	10.9	10.7	11.3
Average no. palindromic repeats	11.6	8.5	8.0	3.0	3.6	1.0	6.5
Average no. of all repeats	90.7	84.2	89.0	139.5	114.0	116.3	103.2

To better understand the potential evolution of gene loss/duplication events and large-scale rearrangements, we compared repeat numbers on DNA sequences near such events. For example, *ycf*1 is a pseudogene in all *Ludwigia* taxa and in *O. curtiflora*, but their totals of repeat numbers in the nearby spacers and in that pseudogene (from seven to 11 repeats) are in the range of the overall family (from six to 43 repeats) (Supporting Information File S8). *Epilobium ulleungensis* has a total of seven repeat elements in the two spacers near its *rps*16 pseudogene, which is in the range of repeat number diversity (from five to seven) for the *rps*16 gene in other *Epilobium* (Supporting Information File S8). *Epilobium* and *Oenothera* species lost one copy of the *ndh*F gene and have one to four repeats, yet they do not have more repeat elements in the nearby spacers than species of the same genera with two copies of that gene, which have one to three repeats (Supporting Information File S8). Neither border of the large 56-kb inversion in *O. biennis* has more repeat elements than found for other *Oenothera* taxa lacking this large inversion (Supporting Information File S8). Accordingly, we found no correlation between repeat numbers and gene loss/duplication events or large-scale rearrangements.

## Discussion

### Phylogenetic inferences in Onagraceae

Our phylogeny of Onagraceae presents an expected intergeneric topology (Fig. 2) (Bult and Zimmer 1993; Conti *et al.* 1993, 1996; Levin *et al.* 2003, 2004; Wagner *et al.* 2007; Berger *et al.* 2016; Maurin *et al.* 2021), with relationships among and within genera largely aligning with the findings of previous studies, except for certain species within *Ludwigia*, *Circaea*, and *Epilobium* (Berry *et al.* 2004; Levin *et al.* 2004; Xie *et al.* 2009; Liu *et al.* 2017; Luo *et al.* 2021; Kundakçi *et al.* 2023).

A published phylogeny of *Ludwigia* based on two nuclear and five chloroplast regions identified six main infrageneric groups––termed clades A1, A2, B1, B2, B3, and B4 (Liu *et al.* 2017). Our plastome-based tree (Fig. 2) indicates that *L. hyssopifolia*, which was previously placed in clade B3 (Liu *et al.*, 2017), is in fact nested within clade B4 and is sister to *L. erecta* (also clade B4). Liu *et al.* (2017) showed that B3 and B4 are well-supported sister clades based on nuclear only sequences, whereas their chloroplast data revealed good support for dividing clade B3 into two subclades and slightly less support for also subividing clade B4 into two subclades. The relationships among these four subclades remained unresolved (Liu *et al.* 2017). Consequently, the relationship between the B3 and B4 clades remains unclear, so additional sampling of *Ludwigia* taxa and further phylogenomic analyses are required.

Moreover, our phylogenetic analysis indicates that both *Circaea alpina* subsp*. caulescens* (Kom.) Tatew. and *C. alpina* subsp*. micrantha* are basal branches of the genus, but these two subspecies do not form a clade (Fig. 2). This outcome suggests that the *C. alpina* L. complex arose at the basal branches of the *Circaea* and is not monophyletic, which is consistent with previous reports (Xie *et al.* 2009; Luo *et al.* 2021). Moreover, given our limited sampling, we could not detect any hybridization events or other complex evolutionary relationships between the only two unilocular species we examined, i.e. *C. alpina* and *C. repens* Wall. ex Asch. & Magnus (Xie *et al.* 2009). Unexpectedly, *C. repens* is nested within bilocular species in our phylogeny (Fig. 2). Boufford (1982; 1990) proposed that unilocular species form a monophyletic group that are nested within bilocular species based on an assessment of morphological and anatomical data. In contrast, the multilocus phylogenies of Xie *et al.* (2009) indicated that bilocular species are a monophyletic group nested within the unilocular species. Though based on a restricted sampling strategy, our phylogeny indicates that neither uniloculares nor biloculares are monophyletic (Fig. 2). Furthermore, our phylogeny points to an earlier origin for the unilocular group relative to the bilocular group (Fig. 2). Further studies involving more samples are needed to untangle inter- and infra-specific relationships, as well as character evolution, in *Circaea*.

Our analysis of *Epilobium* focused exclusively on certain Eurasian taxa in *Epilobium* sect. *Epilobium* (Table 1, Fig. 2), the largest section within this genus (Wagner *et al.* 2007). The infra-sectional relationships we deciphered are mostly consistent with those reported by Luo *et al.* (2021), although we can provide some new insights herein. Taxa exhibiting similar morphologies are generally grouped as sister taxa in our phylogenetic tree (Fig. 2). For instance, *E. minutiflorum* Hausskn. is phylogenetically closest to *E. platystigmatosum*, and *E. tibetanum* is sister to *E. cylindricum*, reflecting their similar morphological characteristics and distributions (Chen *et al.* 2007). However, subspecies of *E. amurense* Hausskn. are not monophyletic according to our phylogeny, so further systematic study is warranted. *Epilobium sikkimense* is basal in our tree and, unexpectedly, does not group with *E. williamsii* P.H. Raven, which it closely resembles in terms of morphology and geographic range (Chen *et al.* 2007). This mismatch between morphological and molecular data might be attributable to interspecific hybridization in sect. *Epilobium* (Seavey and Raven 1977a, 1977b, 1978; Wagner *et al.* 2007) or, alternatively, to parallel evolution or trait transitions that have been reported for other members of the Onagraceae (e.g. Mosquin and Small 1971; Krakos *et al.* 2022). *Epilobium ulleungensis*, endemic to Ulleungdo Island, Korea, was previously proposed to be a natural hybrid between *E. hirsutum* and *E. pyrricholophum* Franch. & Sav. or *E. amurense* subsp*. cephalostigma* (Hausskn.) C.J. Chen, Hoch & P.H. Raven based on morphological data (Chung *et al.* 2017). Albeit with weak support (bs < 50, pp = 0.96), our data suggests that *E. hirsutum*, but not *E. amurense* subsp*. cephalostigma*, is the maternal parent of *E. ulleungensis*.

Previous cytological analyses on sect. *Epilobium* have found that the groups of species in this section can be characterized by chromosome arrangements, which differ from one another by one or more reciprocal chromosome translocations (Seavey and Raven 1977a, 1977b, 1978). The so-called BB chromosome arrangement is most widespread, expecially in Eurasia, and includes all species examined in our study except for *E. platystigmatosum*, which has the apparently unique EE arrangement (the arrangement in *E. ulluengensis* is not known). The placement of *E. platystigmatosum* as sister to *E. minutiflorum* does not necessarily contradict evidence from the nuclear chromosome arrangement. Much more substantial sampling of *Epilobium* species worldwide and nuclear data will be necessary to explore the discrepancies between phylogenetic relationships and morphological traits, as well as to thoroughly resolve the evolutionary history of sect. *Eplilobium*.

### Evolution of Onagraceae plastome, IR, LSC, and SSC sizes

The genera and tribes that branch earliest in Onagroideae possess smaller plastomes, shorter LSC and IR regions, and a longer SSC region than later branching groups (Table 2, Fig. 2). Compared to Onagroideae, Ludwigioideae has a longer LSC and SSC, but a shorter IR. Our data also indicate that plastome size variation increased over the course of Onagroideae evolutionary history, whereas Ludwigioideae exhibits a distinct evolutionary trajectory. These results are consistent with previous morphological and molecular analyses (Raven 1988; Bult and Zimmer 1993; Conti *et al.* 1993, 1996; Levin *et al.* 2003, 2004; Wagner *et al.* 2007; Berger *et al.* 2016; Luo *et al.* 2021; Maurin *et al.* 2021), highlighting the distinct evolutionary histories of these two subfamilies. These plastome features in the Onagraceae corroborate work by Luo *et al.* (2021) and support the second hypothesis of our study regarding IR expansion, with the exception of *Ludwigia* (the only genus in Ludwigioideae).

Our data indicate that IR size is the main contributor to plastome size variation in Onagroideae (r = 0.9843) (Table 3), consistent with findings for many other plant groups (e.g. Wang *et al.* 2017; Rabah *et al.* 2019; Wei *et al.* 2021). Gene number is also positively correlated with Onagroideae plastome size (r = 0.7760) (Table 3). In addition to an expanded IR, some genes in the IR region have duplicated, resulting in an increased number of plastome genes (Table 1, Fig. 2). The co-variation of IR size and gene number in Onagroideae plastomes described herein has also been documented for some other plant groups (e.g. Goulding *et al.* 1996; Sun *et al.* 2016; Rabah *et al.* 2019). Furthermore, repeat number is positively correlated with Onagroideae plastome size (r = 0.7706) (Table 3). Increased repeat number has been reported as an important contributor to plastome size increases in certain plant groups (e.g. Dugas *et al.* 2015; Guo *et al.* 2021). For the four types of repeats we assessed herein, an increase in tandem repeats and a decrease in palindromic repeats are clearly linked to increases in Onagroideae plastome sizes (Table 5). Some previous *Oenothera* studies have postulated that replication slippage in these repeats could be a mechanism potentially affecting plastome sizes (e.g. Wolfson *et al.* 1991; Massouh *et al.* 2016; Huang *et al.* 2021). Additional studies on the evolutionary dynamics of tandem and palindromic repeats will help to elucidate their roles in Onagroideae plastome size increases. Together, our data on Onagroideae provides partial support for our third hypothesis, indicating that IR size, gene number, and repeat number affect plastome size variation.

Interestingly, we found that Ludwigioideae plastome size is not correlated with IR size, gene counts, or repeat counts (Table 3). These results, again, underscore the divergent evolutionary trajectories of the two subfamilies. Unfortunately, our dataset cannot reveal how plastome size evolved in Ludwigioideae, so further species sampling is necessary.

### Genetic variation vs. evolutionary events

We anticipated that DNA sequences of pseudogenes, genes subjected to intron loss, the boundaries of IR regions, and the borders of the 56-kb inversion would exhibit increased repeat numbers and more extensive genetic variations. Indeed, the Onagraceae plastomes we examined exhibit higher average numbers of repeats and greater genetic variation (higher π and lower APSI) in these DNA sequences (Figs. 4-5, Table 4, Supporting Information File S8). Thus, our results expand our knowledge from subsect. *Oenothera* (Greiner, Wang, Rauwolf, *et al.* 2008) to the Onagraceae regarding how repeat elements may be a source of genetic variation in plastomes, as found for other organisms (e.g. Kashi *et al.* 1997; Sperisen *et al.* 2001; Yuan *et al.* 2021), and support our fourth hypothesis.

Furthermore, DNA sequences with high repeat numbers and genetic variations have been used as genetic markers in plant phylogenetic studies (e.g. Yue *et al.* 2009; Liu *et al.* 2020; Machado *et al.* 2020), e.g. the *ycf*2 gene and some spacers, yet we detected no specific evolutionary events in the Onagraceae explaining their extreme variability (Figs. 4-5, Table, Supporting Information File S8). Further studies specifically targeting these DNA sequences separately could provide better insights into the functions and mechanisms underlying their high genetic variations and repeat numbers (Wicke *et al.* 2011; Graham *et al.* 2017).

Although we detected an increase in average repeat numbers in the DNA sequences of evolutionary events in the Onagraceae, comparisons among the sampled taxa indicate that the taxa that have undergone such events do not exhibit more repeats within those DNA sequences. Thus, the evolutionary roles of these events remain enigmatic. If a gene is essential to the plant but becomes pseudogenized in the chloroplast genome, functional copies of this gene might be present in other organellar genomes (Bock 2010; Sanchez-Puerta 2014; Filip and Skuza 2021). Sampling of closely related taxa and at the population level, as well as of artificially mutated individuals of taxa featuring these evolutionary events, could aid in understanding cause-and-effect relationships between these evolutionary events and repeat elements (e.g. Magee *et al.* 2010; Dugas *et al.* 2015; Massouh *et al.* 2016; Williams *et al.* 2019).

Overall, we show that increased numbers of repeat elements and genetic variations can point to possible evolutionary events in Onagraceae plastomes, though the occurrence of such events may not be ubiquitous across all family members.

## Conclusions

Our phylogeny based on 33 Onagraceae plastomes, of which 12 are newly assembled, mostly concurs with previous research on the family, though some clades require further systematic investigation. Onagroideae has undergone an increase in plastome size linked to IR expansion, with Ludwigioideae exhibiting a distinct evolutionary pattern. In addition, high repeat numbers and genetic variations can serve as a marker of DNA regions that have potentially undergone evolutionary events, such as gene loss/gain, with inversion boundaries and IR borders being notable in this respect. This study provides some new insights into plastome evolution in the Onagraceae and we suggest promising avenues for future research focusing on the systematics of *Circaea*, *Epilobium*, and *Ludwigia*, plastome size variation and evolution in Ludwigioideae, and the evolutionary roles of high-repeat regions in plastomes.

## Supporting Information

The following additional information is available in the online version of this article –

plaf025_suppl_Supplementary_Table_S1

## Supporting Information

Supporting Information File S1. The complete chloroplast genomes of all newly assembled Onagraceae samples: (a) *Circaea cordata*, (b) *Epilobium amurense* subsp*. amurense*, (c) *Epilobium platystigmatosum*, (d) *Fuchsia lycioides*, (e) *Ludwigia bonariensis*, (f) *Ludwigia decurrens*, (h) *Ludwigia erecta*, (h) *Ludwigia hyssopifolia*, (i) *Ludwigia lagunae*, (j) *Ludwigia microcarpa*, (k) *Ludwigia perennis*, and (l) *Ludwigia sedoides*. The four rings from outside to inside show the locations of genes, microsatellites, tandem repeats, and forward (red) and reverse (green) repeats, respectively. Genes are color-coded according to their function, as per the legend. Genes inside the first ring are in clockwise directions, whereas genes on the outside are in counterclockwise directions.

Supporting Information File S2. Results of the Shimodaira-Hasegawa (SH) and Kishino–Hasegawa (KH) tests, showing that tree topologies generated from different algorithms are consistent.

Supporting Information File S3. Bayesian 50% majority rule consensus tree based on 33 Onagraceae and three Lythraceae plastomes. In cases where branches are not fully supported, posterior probabilities (pp)/bootstrapping (bs) values are shown. Hyphens represent if the pp or bs value is less than 0.70 or 50, respectively. The scale bar denotes the branch length.

Supporting Information File S4. The final alignment, partitions, and tree files.

Supporting Information File S5. A list of annotated unique genes, gene functions, and introns for studied Onagraceae plastomes.

Supporting Information File S6. A pairwise syntenic dotplot between *Ludwigia microcarpa* and *Oenothera biennis*, generated in mVISTA, shows that the latter taxon has a large 56-kb inversion (red arrow). The inverted repeat (IR) (black arrow) is also indicated.

Supporting Information File S7. The nucleotide variations (Pi; π) of the complete chloroplast genomes within *Oenothera* based on the a priori alignment (pAL). Nine regions with high π are denoted with letters. Genes, spacers, quadripartite structure boundaries, and evolutionary events found in these regions are listed in Table 4. The plastomes of *Oenothera curtiflora* (a) and *Oenothera biennis* (b) are shown here as references to indicate the positions and direction of genes on the pAL.

Supporting Information File S8. (a) Numbers and distributions of repeat elements in the plastome of each studied sample in the Onagraceae. (b) Average repeat number at each gene and spacer in the Onagraceae plastome. (Excel file)

Supporting Information File S9. Detailed results of the repeat element analysis. The files are available at DOI: 10.6084/m9.figshare.26421670.

## Data Availability

The newly generated plastome sequences have been submitted to NCBI (https://www.ncbi.nlm.nih.gov/). GenBank accession numbers are shown in Table 1.
