# Supplementary material for: Novel perspectives on plastome evolution in Onagraceae
Source: AoB Plants. 2025 Apr 24;17(3):plaf025. doi: 10.1093/aobpla/plaf025 (PMC12190799; doi:10.1093/aobpla/plaf025)
Supplement: plaf025_suppl_Supplementary_Table_S1 [file plaf025_suppl_supplementary_table_s1.zip › plaf025_Suppl_data/SI.all_revisedMS_tracked.pdf]

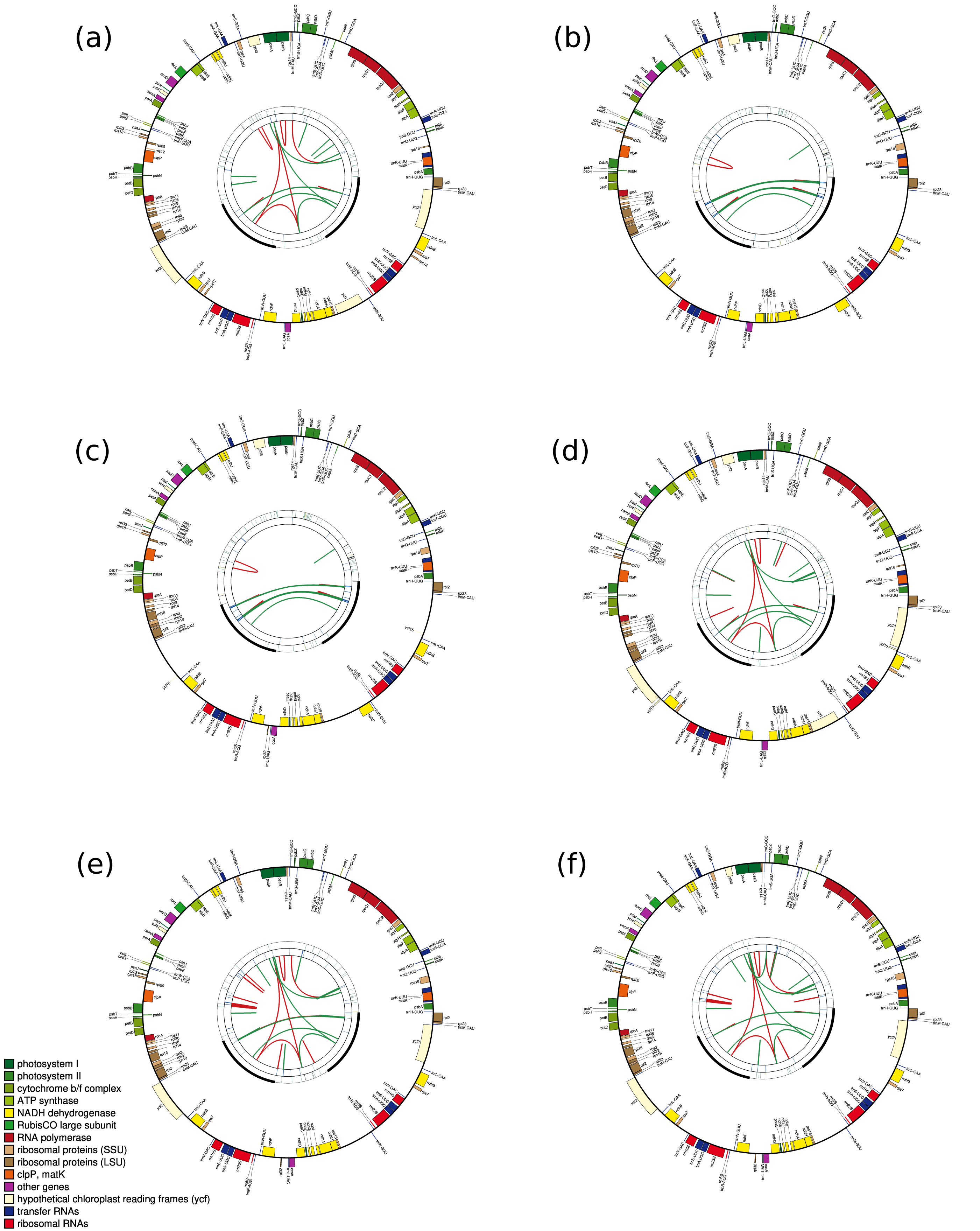
(a)
(b)
(c)
(d)
(e)
(f)
photosystem I
photosystem II
cytochrome b/f complex
ATP synthase
NADH dehydrogenase
RubisCO large subunit
RNA polymerase
ribosomal proteins (SSU)
ribosomal proteins (LSU)
clpP, matK
other genes
hypothetical chloroplast reading frames (ycf)
transfer RNAs
ribosomal RNAs

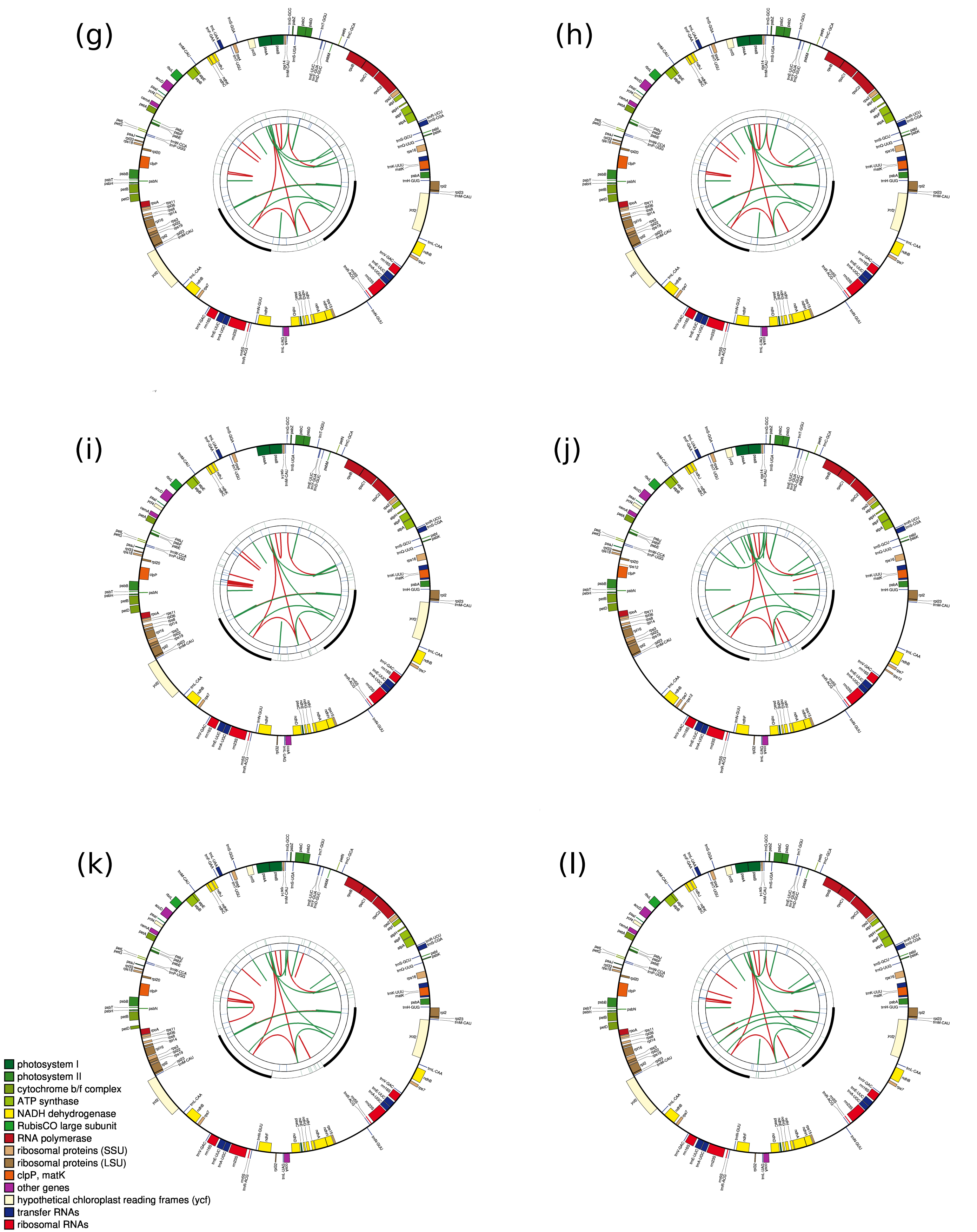
(g)
(h)
(i)
(j)
(k)
(l)
photosystem I
photosystem II
cytochrome b/f complex
ATP synthase
NADH dehydrogenase
RubisCO large subunit
RNA polymerase
ribosomal proteins (SSU)
ribosomal proteins (LSU)
clpP, matK
other genes
hypothetical chloroplast reading frames (ycf)
transfer RNAs
ribosomal RNAs

Supporting Information File S1. The complete chloroplast genomes of all newly assembled Onagraceae samples: (a) *Circaea cordata*, (b) *Epilobium amurense* subsp. *amurense*, (c) *Epilobium platystigmatosum*, (d) *Fuchsia lycioides*, (e) *Ludwigia bonariensis*, (f) *Ludwigia decurrens*, (h) *Ludwigia erecta*, (h) *Ludwigia hyssopifolia*, (i) *Ludwigia lagunae*, (j) *Ludwigia microcarpa*, (k) *Ludwigia perennis*, and (l) *Ludwigia sedoides*. The four rings from outside to inside show the locations of genes, microsatellites, tandem repeats, and forward (red) and reverse (green) repeats, respectively. Genes are color-coded according to their function, as per the legend. Genes inside the first ring are in clockwise directions, whereas genes on the outside are in counterclockwise directions.

Formatted: Width: 21.59 cm, Height: 27.94 cm

Supporting Information File S2. The results of Shimodaira-Hasegawa (SH) and Kishino–Hasegawa (KH) tests show the topological consistencies among different algorithms for phylogeny reconstruction.

p-values	**Algorithm for phylogeny reconstruction**	
	ML	BI
KH test	0.8903	Best tree
SH test	0.4495	Best tree

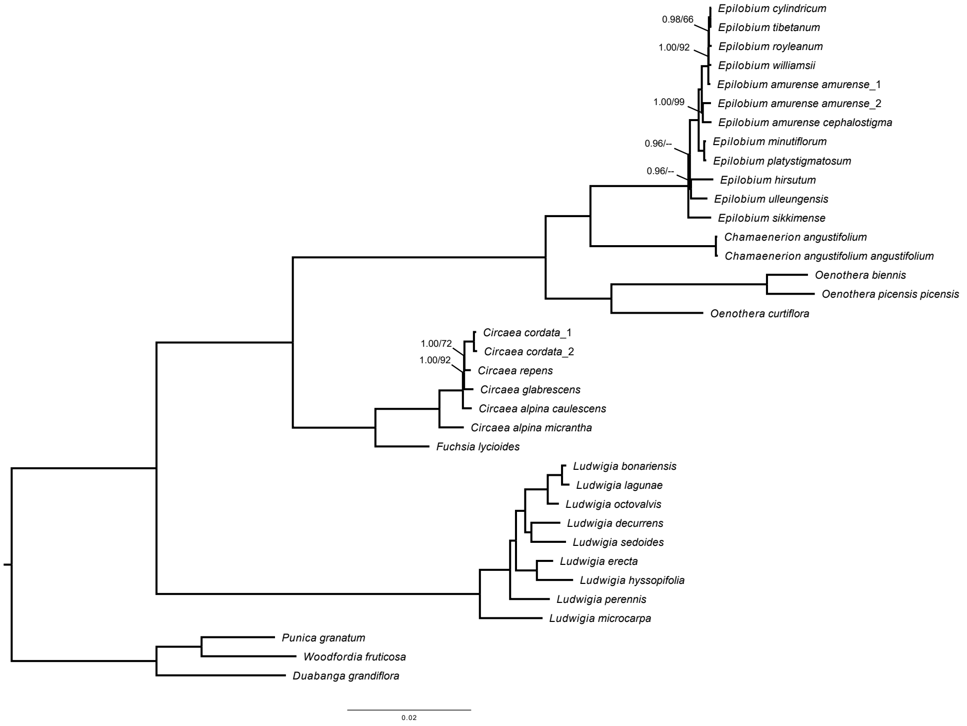

Supporting Information File S3. Bayesian 50% majority rule consensus tree based on 33 Onagraceae and three Lythraceae plastomes. In cases where branches are not fully supported, posterior probabilities (pp)/bootstrapping (bs) values are shown. Hyphens represent if the pp or bs value is less than 0.70 or 50, respectively. The scale bar denotes the branch length.

Supporting Information File S4. The final alignment, partitions, and tree files. (Large text file)

Deleted: ¶
¶
¶

Supporting Information File S5. A list of unique genes, gene functions, and introns annotated on studied Onagraceae plastomes.

Gene function: Genes

Photosynthesis

Photosystem I: *psaA, psaB, psaC, psaI, psaJ, ycf3, ycf4*

Photosystem II: *psbA, psbB, psbC, psbD, psbH, psbI, psbJ, psbK, psbL, psbM, psbT, psbZ*

Cytochrome b6/f complex: *petA, petB, petD*[#]*, petG, petL, petN, psbE, psbF*

ATP synthase: *atpA, atpB, atpE, atpF, atpH, atpI*

NADH dehydrogenase: *ndhA, ndhB, ndhC, ndhD, ndhE, ndhF, ndhG, ndhH, ndhI, ndhJ, ndhK*

RubisCo large subunit: *rbcL*

Self replication

RNA polymerase: *rpoA, rpoB, rpoC1, rpoC2*

Ribosomal proteins (Large subunit): *rpl2, rpl14, rpl16, rpl20, rpl22, rpl23, rpl32, rpl33, rpl36*

Ribosomal proteins (Small subunit): *rps2, rps3, rps4, rps7, rps8, rps11, rps12, rps14, rps15, rps16*[$]*, rps18, rps19*

Proteins of unknown function: *ycf1*[@]*, ycf2*

Other proteins: *accD*, *ccsA, cemA, clpP, matK, pbf1*

Transfer RNAs: *trnA-UGC, trnC-GCA, trnD-GUC, trnE-UUC, trnF-GAA, trnfM-CAU II, trnG-GCC, trnG-UCC, trnH-GUG, trnI-CAU, trnI-GAU, trnK-UUU, trnL-CAA, trnL-UAA, trnL-UAG, trnM-CAU, trnN-GUU, trnP-UGG, trnQ-UUG, trnR-ACG, trnR-UCU, trnS-GCU, trnS-GGA, trnS-UGA, trnT-GGU, trnT-UGU, trnV-GAC, trnV-UAC, trnW-CCA, trnY-GUA*

Ribosomal RNAs: *rrn4.5, rrn5, rrn16, rrn23*

Introns

atpF intron, *clpP* intron 1*, *clpP* intron 2*, *ndhA* intron, *ndhB* intron, *ndhB* intron, *petB* intron, *petD* intron, *rpl2* intron, *rpl2* intron, *rpl16* intron, *rpoC1* intron, *rps12* intron, *rps12* intron, *rps16* intron, *trnA-UGC* intron, *trnA-UGC* intron, *trnG-UCC* intron, *trnI-GAU* intron, *trnI-GAU* intron, *trnK-UUU* intron, *trnL-UAA* intron, *trnV-UAC* intron, *ycf3* intron 1, *ycf3* intron 2

Note:

Pseudogene in *Ludwigia hyssopifolia*

* Intron loss in *Oenothera biennis* and *O. picensis picensis*

$ Pseudogene in *Epilobium ulleungensis*

@ Pseudogene in all *Ludwigia* samples and *Oenothera curtiflora*

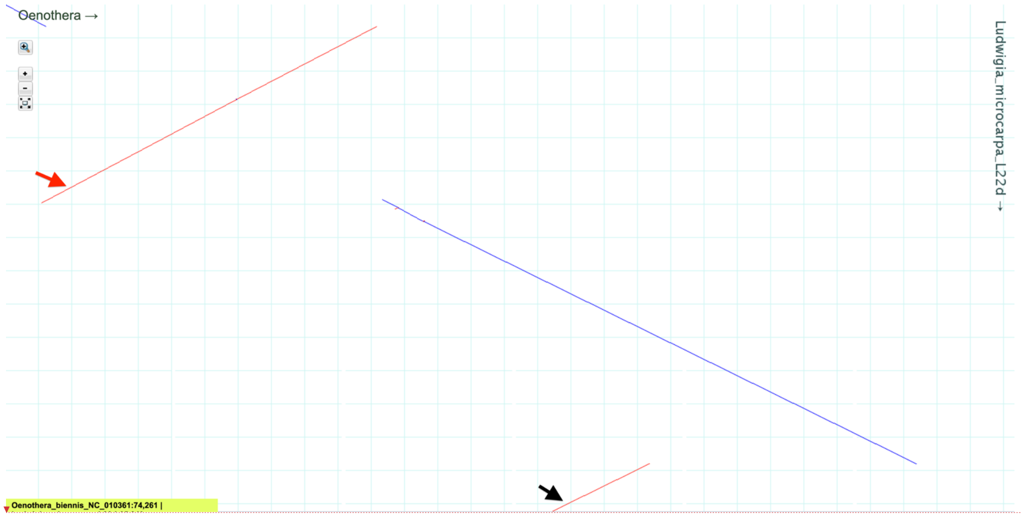

Supporting Information File S6. A pairwise syntenic dotplot between *Ludwigia microcarpa* and *Oenothera biennis*, generated in mVISTA, shows that the latter taxon has a large 56-kb inversion (red arrow). The inverted repeat (IR) (black arrow) is also indicated.

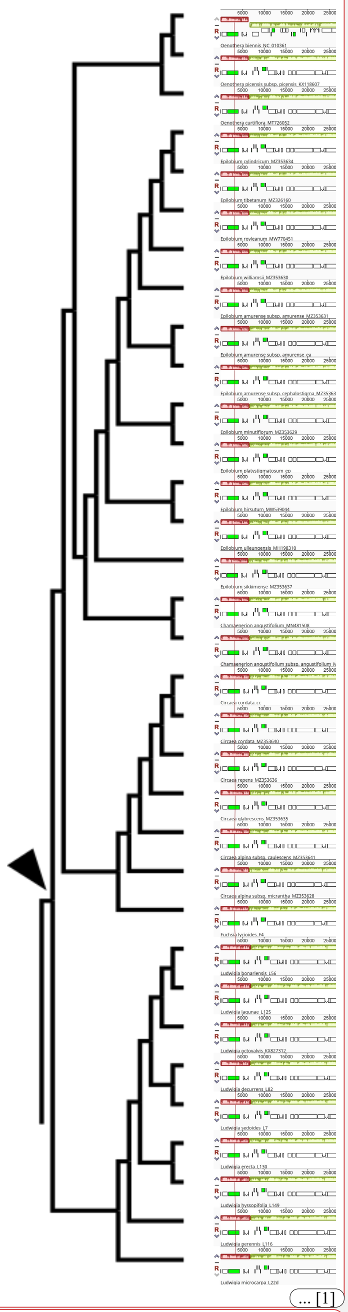

Deleted: ... [1]

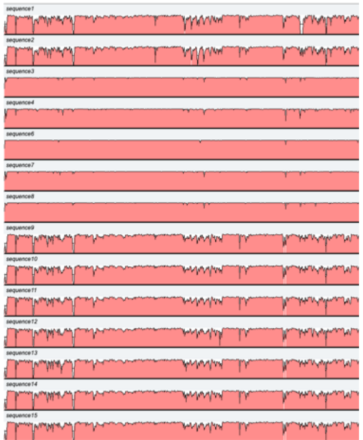

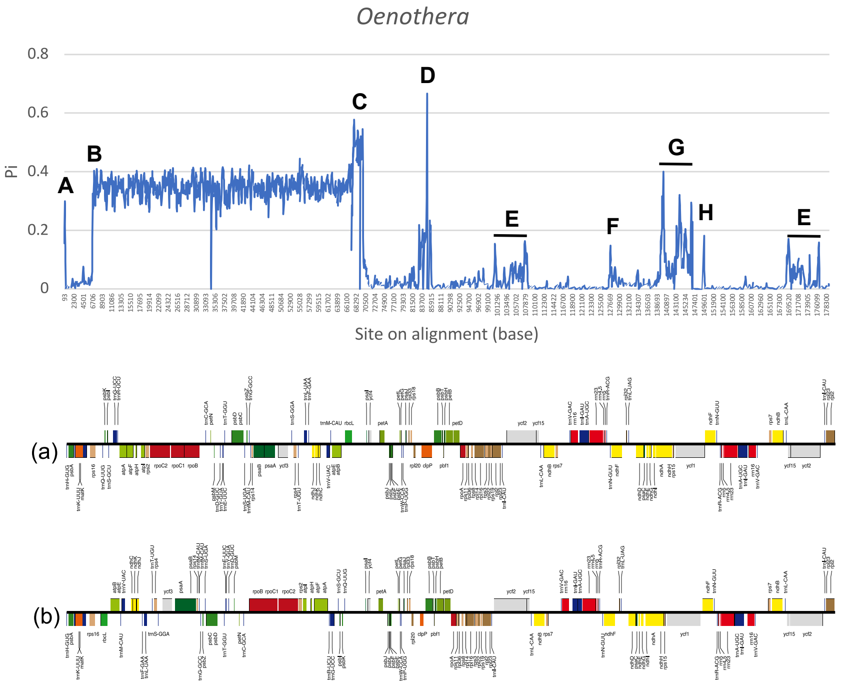

Supporting Information File S7. The nucleotide variations (Pi; π) of the complete chloroplast genomes within *Oenothera* based on the *a priori* alignment (pAL). Nine regions with high π are denoted with letters. Genes, spacers, quadripartite structure boundaries, and evolutionary events found in these regions are listed in Table 4. The plastomes of *Oenothera curtiflora* (a) and *Oenothera biennis* (b) are shown here as references to indicate the positions and direction of genes on the pAL.

Deleted: S9

Deleted: Pi

Deleted: Pi values

Deleted: as in Fig. 4

Deleted: ¶

Supporting Information File S8. (a) Numbers and distributions of repeat elements in the plastome of each studied sample in the Onagraceae. (b) Average repeat number at each gene and spacer in the Onagraceae plastome. (Excel file)

Supporting Information File S9. Detailed results of the repeat element analysis. The files are available at DOI: 10.6084/m9.figshare.26421670.

Deleted: Supporting Information File S10. Twenty genes or spacers with the lowest average pairwise sequence identities (APSI) in each genus, subfamily, and family. Regions exhibiting high nucleotide variation (Pi) are denoted with letters, as in Fig. 4. Seven additional spacers are denoted: A'. *rpl*2-*trn*H-GUG spacer, a boundary between the reverted repeat and large single copy (IR-LSC); D'. *rps*12 exon 1-*clp*P spacer; E'. *trn*I-CAU-*ycf*2 spacer; F'. *rpl*32-*trn*L-UAG spacer; K. *rpl*22-*rps*19 spacer; L. *trn*R-UCU-*atp*A spacer; and M. *ccs*A-*ndh*D spacer.

Family Onagraceae ... [3]

Deleted:

Page Break

Supporting Information File S11. Average pairwise sequence identities (APSI) of each chloroplast gene and spacer in (a) *Oenothera*, (b) *Epilobium*, (c) *Chamaenerion*, (d) *Circaea*, (e) subfamily Onagroideae, (f) *Ludwigia* or subfamily Ludwigioideae, and (g) family Onagraceae. Genes or spacers with relatively low APSI are denoted with letters. The positions (bp) of each gene and spacer on the complete chloroplast genome are shown relative to those of the *Fuchsia lycioides* plastome illustrated below panels (c) and (g). The letters are as per Fig. 4. Seven additional spacers are denoted: A'. *rpl*2-*trn*H-GUG spacer, a boundary of the reverted repeat and large single copy (IR-LSC); D'. *rps*12 exon 1-*clp*P spacer; E'. *trn*I-CAU-*ycf*2 spacer; F'. *rpl*32-*trn*L-UAG spacer; K. *rpl*22-*rps*19 spacer; L. *trn*R-UCU-*atp*A spacer; and M. *ccs*A-*ndh*D spacer. Note that only one reverted repeat is displayed.

Page Break

Supporting Information File S12. Average pairwise sequence identities (APSI) for each chloroplast gene and spacer in genera *Oenothera*, *Epilobium*, *Chamaenerion*, *Circaea*, *Ludwigia*, subfamily Onagroideae, and family Onagraceae. (Excel file)

Supporting Information File S13. (a) Numbers and distributions of repeat elements in the plastome of each studied sample in the Onagraceae. (b) Average repeat number at each gene and spacer in the Onagraceae plastome. (Excel file)

Deleted: S14

Deleted: ... [4]

Page 5: [1] Deleted **Microsoft Office User** **3/17/25 11:46:00 AM**

Page 5: [2] Deleted **Microsoft Office User** **3/17/25 11:46:00 AM**

Page 7: [3] Deleted **Microsoft Office User** **3/17/25 11:50:00 AM**

Page 7: [4] Deleted **Microsoft Office User** **3/20/25 8:04:00 PM**
